# Compuflo®-Assisted Training vs Conventional Training for the Identification of the Ligamentum Flavum with an Epidural Simulator: A Brief Report

**DOI:** 10.1155/2019/3804743

**Published:** 2019-09-12

**Authors:** Emanuele Capogna, Alessandra Coccoluto, Giovanni Gibiino, Angelica Del Vecchio

**Affiliations:** ^1^European School of Obstetric Anesthesia (EESOA), Maternal Neonatal Simulation Centre, Rome, Italy; ^2^Department of Anesthesiology, Casa di Cura Gibiino, Catania, Italy

## Abstract

The ability of recognizing the ligamentum flavum is the first, crucial, important skill to teach novices when they are learning the epidural technique. The aim of this preliminary prospective study was to evaluate whether the Compuflo® Epidural instrument may help inexperienced trainees to better identify the ligamentum flavum during an epidural simulator training session. Compuflo® reduced the total number of attempts to identify the ligamentum flavum and increased three fold the chance to identify it at the first attempt during a simulator-assisted training module, making this identification easier for inexperienced trainees. This trial is registered with NCT03812926.

## 1. Introduction

The correct performance of an epidural block for the provision of regional anesthesia is considered difficult for 1st-year residents with a learning curve of 60–90 attempts to reach a satisfactory level of proficiency, the longest among all the regional anesthesia techniques [1, 2].

The first and important skill that any trainee has to face when he/she is going to learn the epidural technique is the ability of recognizing the ligamentum flavum before proceeding with the loss of resistance technique. For the novice, it may be difficult to differentiate any increase of resistance encountered by the epidural needle especially in obstetric patients who may have softer ligaments.

The CompuFlo® Epidural instrument (Milestone Scientific, Inc. Livingston, New Jersey, USA) is a computer-controlled drug delivery system capable of distinguishing different tissue types by providing continuously real-time “exit-pressure” data at the needle tip and that has been validated as a useful tool to detect the epidural space [3–6]. This instrument uses an algorithm to determine the pressure at the tip of the needle via a continuous fluid path. The pressure is a feed-back loop and controller to the system, thus regulating the electromechanical motor which controls flow-rate and the fluid dispensed by the system. An audible and visual graphic of exit-pressure is provided to the health care practitioner enabling the operator to focus on the injection site [3, 4]. We previously described a new custom-made simulator, demonstrating its realism by the similarity between the simulator and human pressure' curves objectively obtained with the CompuFlo® Epidural instrument [5].

The aim of this prospective, single blinded study was to evaluate whether the Compuflo® Epidural instrument may help inexperienced trainees to better identify the ligamentum flavum during an epidural simulator training course.

## 2. Methods

Ethical approval was waived by the Local Research Ethics Committee, and it was sought and deemed not necessary because no patients were involved in the study. The study was registered at ClinicalTrials.gov with the identification number NCT03812926.

After having obtained their written informed consent, we enrolled 60 trainees who had never previously performed an epidural block. After having had a standardized learning module on epidural anatomy and technique, each trainee practiced the epidural procedure on the epidural simulator. All the trainees were unaware of the purpose of this study which they had voluntary joined.

In accordance with our standard teaching method, the trainees were asked to insert the Tuohy needle until they felt an increase of resistance which they believed to be the ligamentum flavum. An expert instructor immediately checked this hypothesis by attaching a syringe to the trainee's needle and by performing the loss of resistance technique. In this way, the instructor was able to confirm that the needle had indeed passed through the ligamentum flavum.

In the case of a false increase of resistance, the instructor allowed the trainee to repeat the procedure up to five times.

**Figure 1:**
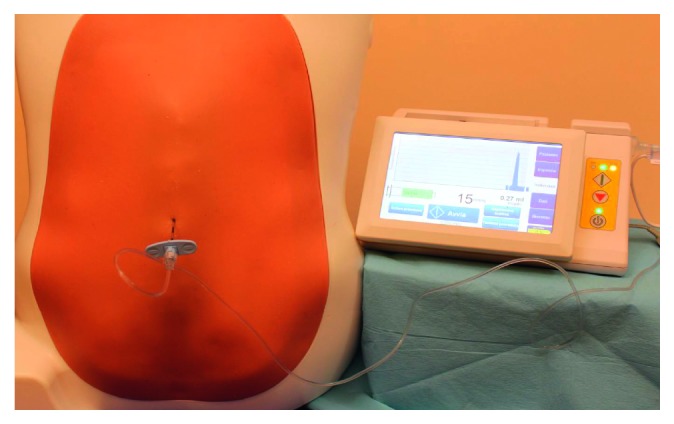
Study settings: the Compuflo® epidural instrument and the epidural simulator.

Each trainee repeated the procedure with the Compuflo® instrument (Figure 1).

Using the CompuFlo®, the entry of the needle into the ligamentum flavum is indicated by a great increase in pressure on the visual display with a simultaneous increase of the pitch of the audible tone, while the entry of the needle into the epidural space results in a brisk drop in pressure and a distinct fall in the tone of the audio output. A drop in pressure sustained for more than 5 seconds is deemed to be consistent with entry into the epidural space [3–6].

After having received instruction on the use of Compuflo® instrument, the trainees were asked to insert the Tuohy needle until they observed a great increase in pressure on the instrument's display with a simultaneous increase of the pitch of the audible tone, both recognized signs of the entry of the needle into the ligamentum flavum. An expert instructor then checked the position of the needle by performing a Compuflo®-assisted loss of resistance technique. In the case of a false increase of resistance, the instructor allowed the trainee to repeat the procedure up to five times.

The number of attempts was noted.

The power analysis required a sample size of 60 observations to set 80% test power and a 95% significance. The unpaired *T* test was used to evaluate the differences in the number of epidural attempts. A logistic regression model was used to correlate the “success at the first attempt ratio” and the technique used.

## 3. Results

**Figure 2:**
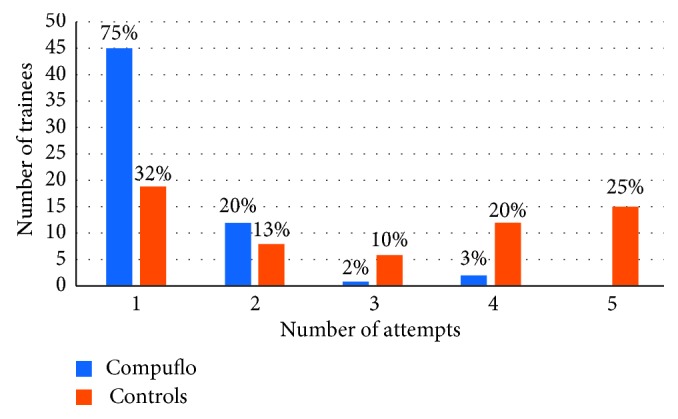
Number (%) of trainees plotted vs the number of attempts needed to correctly identify the ligamentum flavum.

All the trainees completed the study. The number (median, range) of attempts was 1 (1-1) in the Compuflo®-assisted technique series and 3 (1–4) in the control series (*P* < 0.05). In Figure 2, the number (%) of trainees plotted vs the number of attempts needed to correctly identify the ligamentum flavum is reported. The odds ratio to identify the ligamentum flavum at the first attempt was 2.82.

## 4. Discussion

In clinical practice, the epidural needle is most frequently introduced, particularly by trainees, in the lumbar area to a depth of approximately 2-3 cm due to the fear of inadvertent dural puncture. This way the needle may be located somewhere between the soft tissues, and it is therefore crucial for the beginner to become able to recognize the feeling of the increased resistance due to the needle's initial penetration into the ligamentum flavum.

In this study, Compuflo®-assisted training reduced the number of attempts to correctly identify the ligamentum flavum and made this identification easier for inexperienced trainees. Indeed, Compuflo® increased three fold the chance to identify the ligamentum flavum at the first attempt during a simulator-assisted training module.

An important issue linked to a more certain and easier identification of the feeling of resistance during the epidural procedure is that it may lead to less epidural attempts and/or needle reinsertions, with a reduced risk of accidental dural puncture especially when the procedure is performed by trainees.

We are aware that this preliminary study represents the first, initial, but necessary step towards a more complete and extensive investigation on the potential contribution of Compuflo®-assisted training in teaching the epidural technique. We believe our result may encourage greater use of this device and its more extensive routine evaluation, in order to determine its contribution to a simpler and more reliable learning of the epidural technique.

## Data Availability

The data used to support the findings of this study are available from the corresponding author upon request.
